# Supplementary material for: Photo-induced stress relaxation in reconfigurable disulfide-crosslinked supramolecular films visualized by dynamic wrinkling
Source: Nat Commun. 2022 Dec 2;13:7434. doi: 10.1038/s41467-022-35271-9 (PMC9718802; doi:10.1038/s41467-022-35271-9)
Supplement: Supplementary file 1 — Supplementary Information File [file 41467_2022_35271_MOESM1_ESM.pdf]

Supplementary Information

Photo-induced stress relaxation in reconfigurable disulfide-crosslinked supramolecular films visualized by dynamic wrinkling

Shuzhen Yan[1], Kaiming Hu[2], Shuai Chen[1], Tiantian Li[1], Wenming Zhang[2] *, Jie Yin[1] and Xuesong Jiang[1] *

[1]School of Chemistry & Chemical Engineering, Frontiers Science Center for Transformative Molecules, State Key Laboratory for Metal Matrix Composite Materials, Shanghai Jiao Tong University, Shanghai 200240, P. R. China

[2]State Key Laboratory of Mechanical Systems and Vibration, School of Mechanical Engineering, Shanghai Jiao Tong University, Shanghai 200240, P. R. China

E-mail: ponygle@sjtu.edu.cn, wenmingz@sjtu.edu.cn

Supplementary Figures

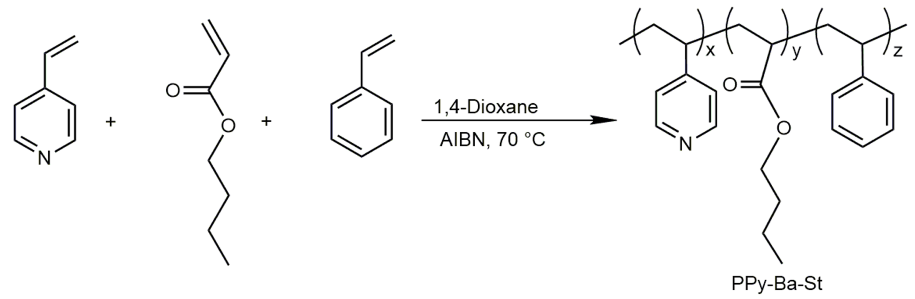

Supplementary Fig. 1. The synthesis route to light-sensitive copolymer PPy-Ba-St.

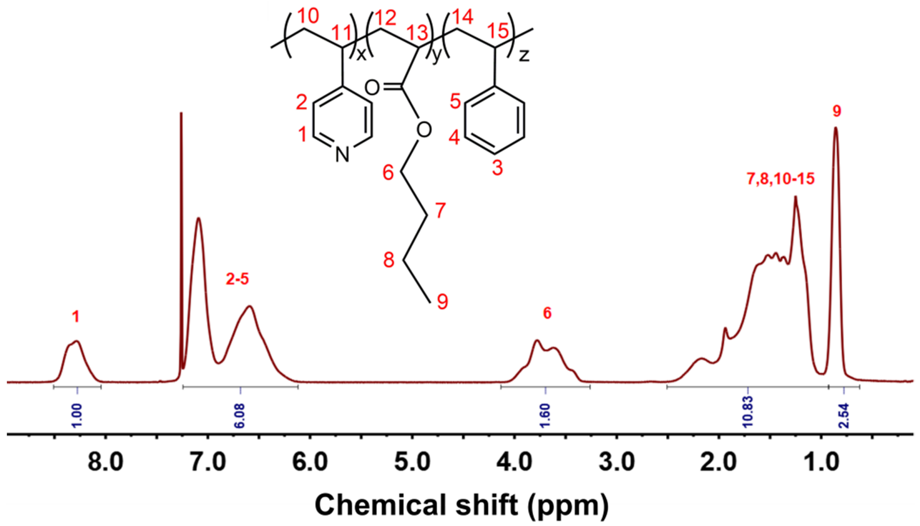

Supplementary Fig. 2. ^{1}H NMR spectrum of PPy-Ba-St in $CDCl_3$ solution at 298 k.

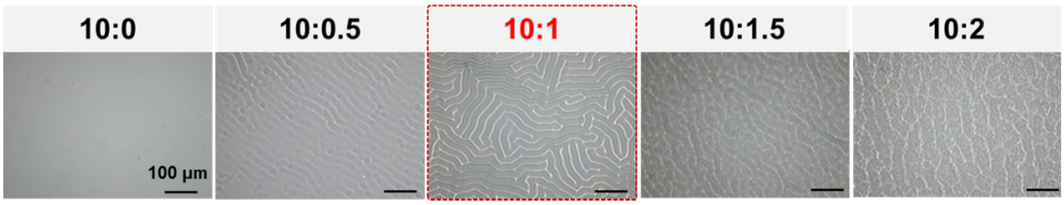

Supplementary Fig. 3. Evolution of the wrinkled morphology. The different concentration of DTNB in the polymer, 10:0, 10:0.5, 10:1, 10:1.5; 10:2. Scale bars: 100 μm.

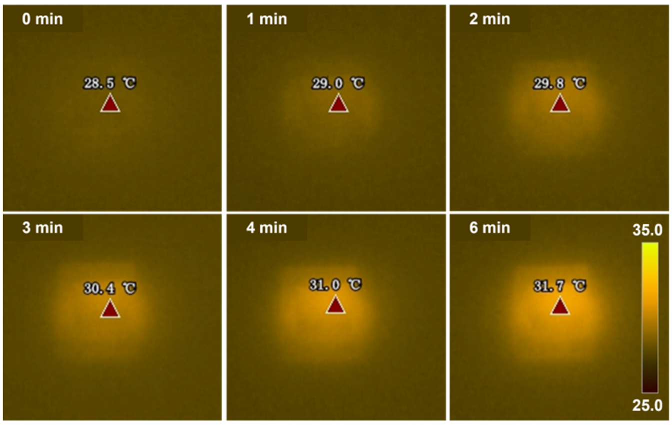

Supplementary Fig. 4. Temperature variation of PDMS elastomer. The sample irritated by 365 nm UV light (15 mW/cm^2).

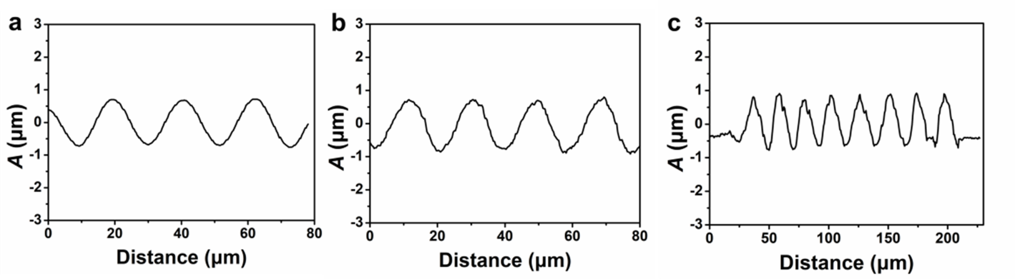

Supplementary Fig. 5. The corresponding line graphs of the marked area by red line in Fig. 1e. a The random wrinkles before UV light exposure. **b** The 1D ordered wrinkles in unexposed area after UV light exposure. **c** The wrinkled pattern in unexposed area after heating/cooling treatment. Source data are provided as a Source Data file.

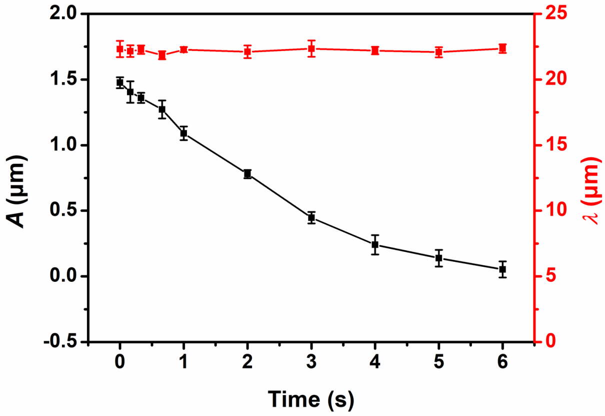

Supplementary Fig. 6. Physical properties of the wrinkle patterns. The corresponding amplitude (A, black line) and wavelength (λ, red line) of the wrinkles as a function of 365 nm UV light exposure. Error bars represent the standard deviations of three independent data. Source data are provided as a Source Data file.

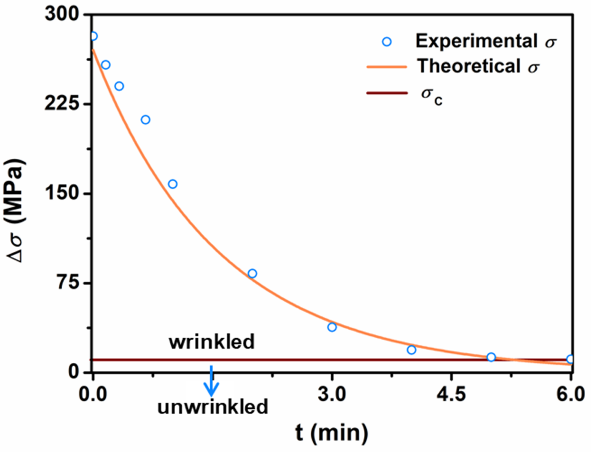

Supplementary Fig. 7. Stress distributions in the top supramolecular network. The experimental stress σ (blue circle), theoretical stress σ (yellow line), and critical stress σ_c (pink line) of the wrinkled surface upon 365 nm light irradiation for different time. Source data are provided as a Source Data file.

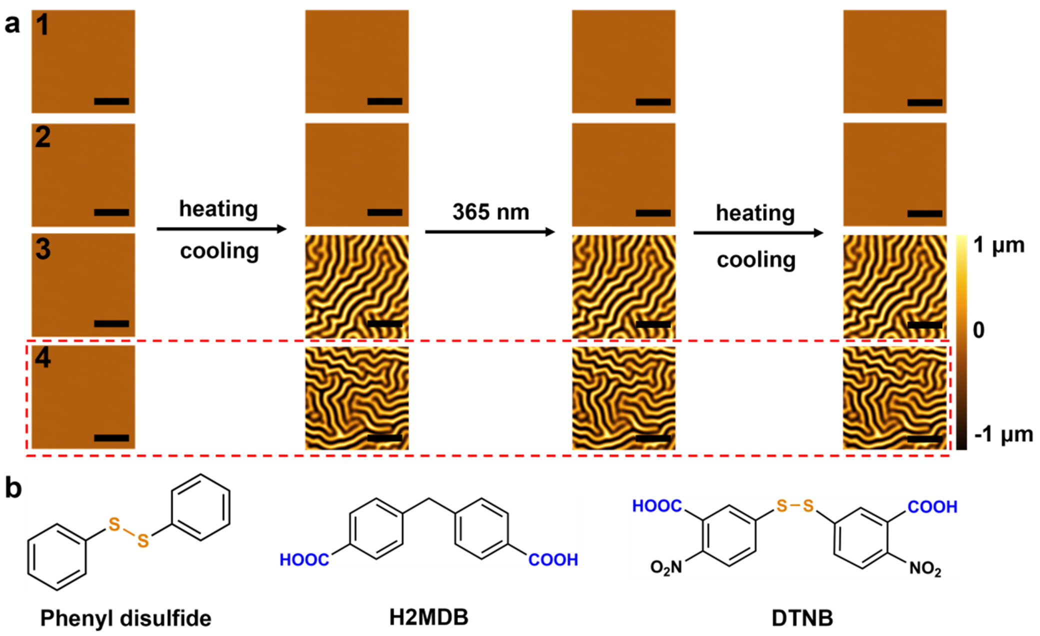

Supplementary Fig. 8. The control samples with different components of the top film under heating/cooling treatment. a LSCM images of (1) PPy-Ba-St, (2) PPy-Ba-St@Phenyl disulfide, (3) PPy-Ba-St@H2MDB and (4) PPy-Ba-St@DTNB bilayer wrinkling systems upon 365 nm UV light irradiation and heating/cooling treatment, respectively. **b** Molecular chemical structural formulas of the bilayer systems. Scale bar: 100 μm. Source data are provided as a Source Data file.

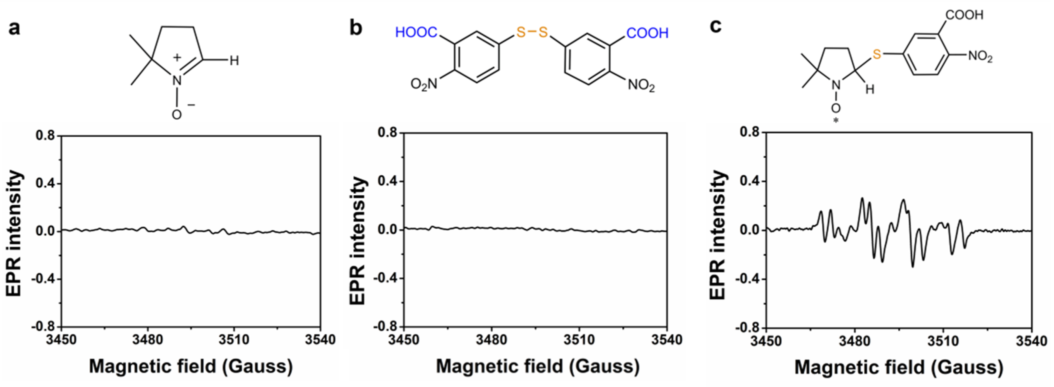

Supplementary Fig. 9. Generation of thiol radical. EPR spectra of **a** DMPO, **b** DTNB, and **c** 1 mM DTNB dissolved in THF in the presence of DMPO (0.88 M) under 365 nm UV light. Source data are provided as a Source Data file.

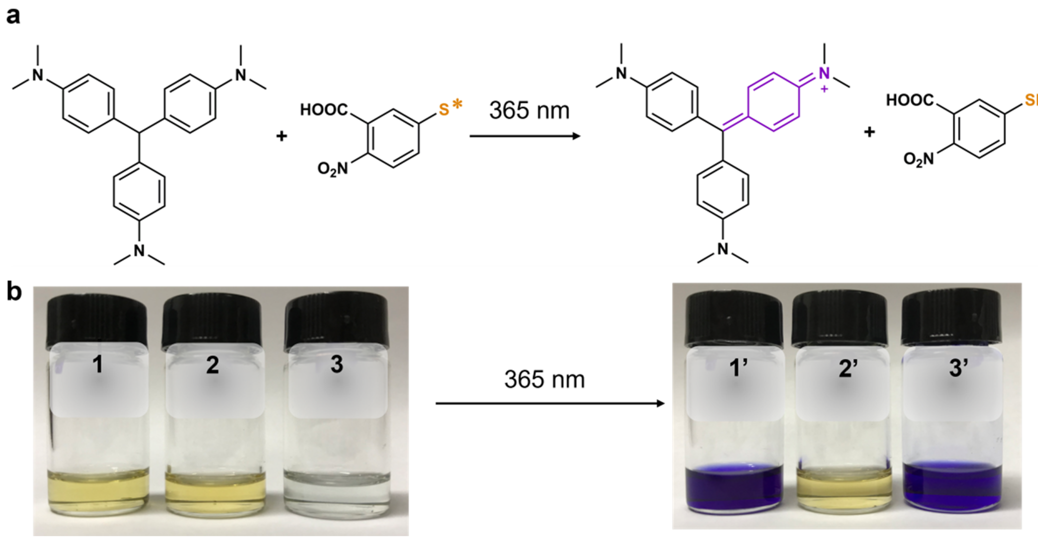

Supplementary Fig. 10. Generation of thiol radical in the photoinduced disulfide exchange reaction. a The generation route of thiol radicals under 365 nm UV light in the presence of LCV (0.42 mM). **b** Optical images of (1) PPy-Ba-St@DTNB, (2) PPy-Ba-St, (3) DTNB dissolved in THF with LCV.

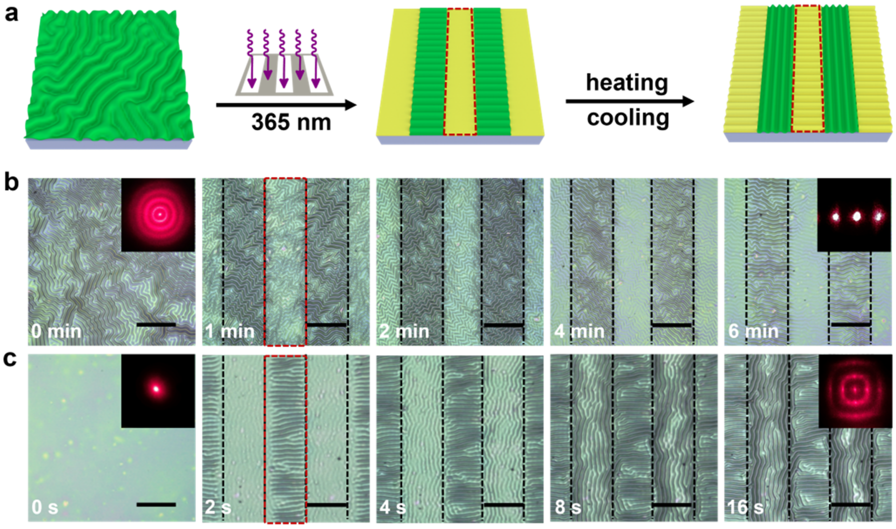

Supplementary Fig. 11. Large-area fabrication of the light-controlled wrinkle patterns by a lithography-compatible exposure strategy. a Schematic illustration of the tunable wrinkle, which selectively exposed to 365 nm UV light based on (PPy-Ba-St@DTNB)/PDMS bilayer system. 2D LSCM images of the **b** erasure and **c** regeneration process of wrinkles through a striped mask. Scale bars: 200 μm.

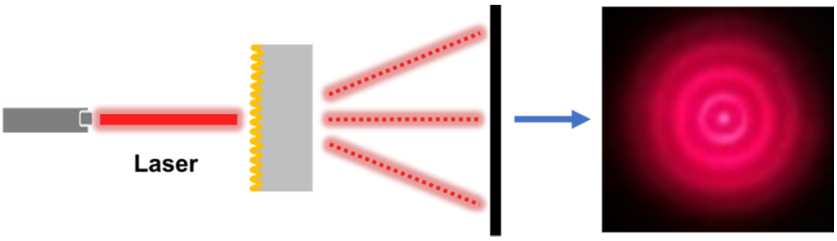

Supplementary Fig. 12. Light diffraction patterns. Schematic demonstration of the diffraction patterned grating based on microstructures.

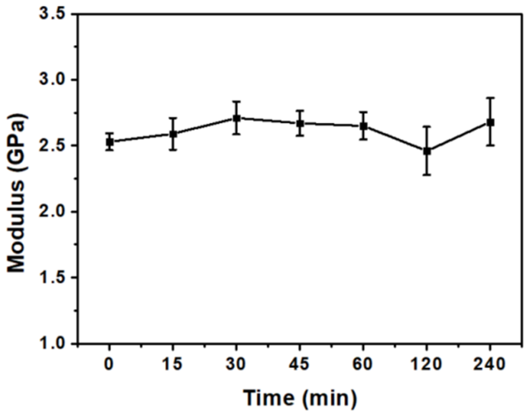

Supplementary Fig. 13. Young's moduli of the top film. The corresponding modulus of the

wrinkle versus illumination time in exposed region, determined by the atomic force microscopy (AFM). Error bars represent the standard deviations of three independent data. Source data are provided as a Source Data file.

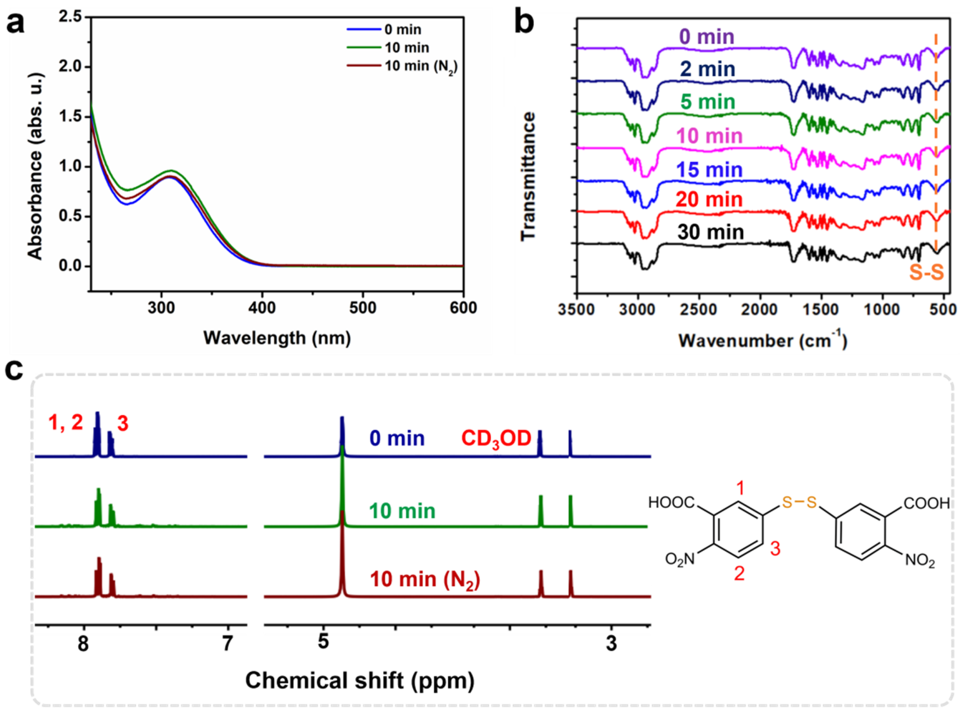

Supplementary Fig. 14. Photostability of DTNB and PPy-Ba-St@DTNB. a UV spectra and ^{1}H NMR spectra **(c)** of DTNB for 10 min of 365 nm UV light with or without N_2. **b** FTIR spectra of 14 wt.% PPy-Ba-St@DTNB film for different irradiation times of 365 nm UV light. Source data are provided as a Source Data file.

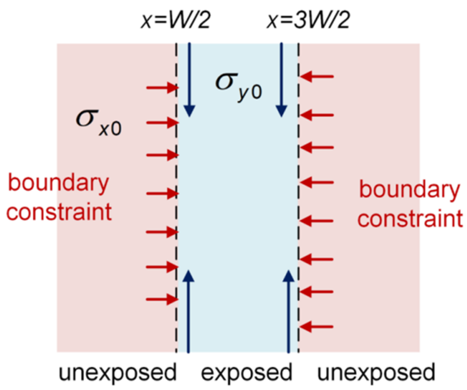

Supplementary Fig. 15. Stress distribution of the exposed region. 1D stress relaxation is triggered by the disulfide bond exchange-induced stress relaxation of the unexposed region.

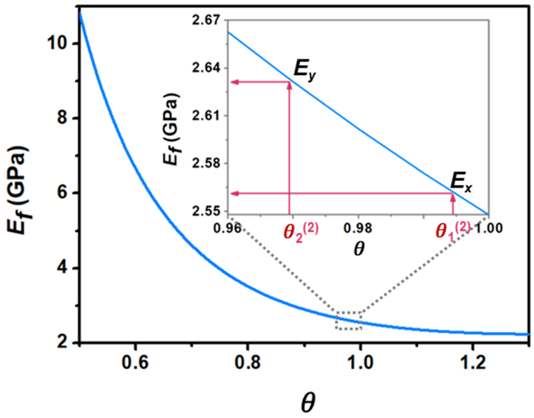

Supplementary Fig. 16. The elastic modulus of the deformed polymer network in the exposed regions. Source data are provided as a Source Data file.

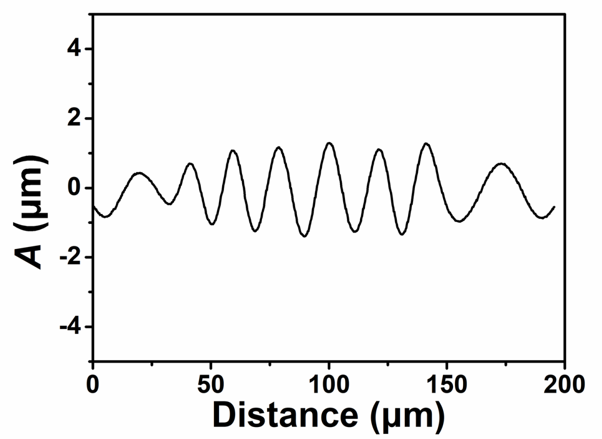

Supplementary Fig. 17. The corresponding line graphs of the marked area by the red line in Fig. 4e. Source data are provided as a Source Data file.

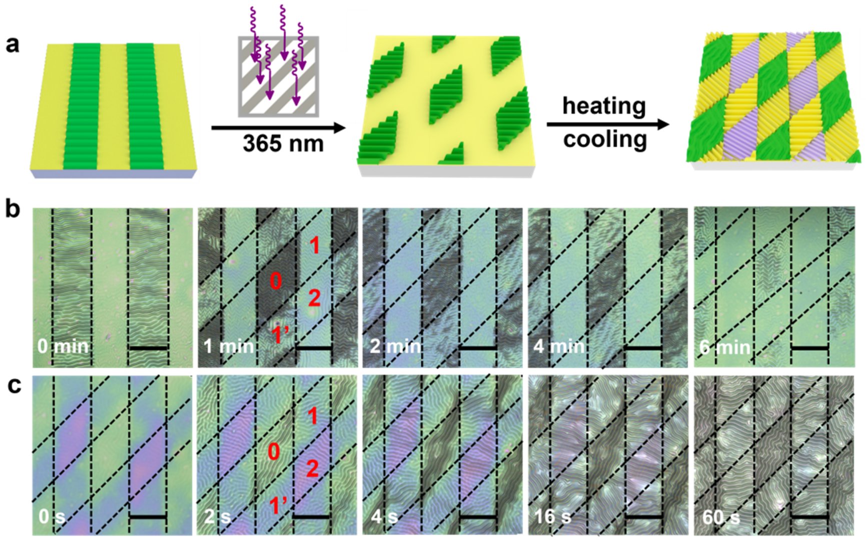

Supplementary Fig. 18. Sequential exposure strategy for tuning alternative wrinkle microstructures, a continuous second selective UV radiation with 45° inclined strip mask. a

Schematic illustration of the tunable wrinkle. 2D LSCM images of the **b** erasure and **c** regeneration behavior of wrinkles that was selectively exposed to 365 nm UV light through a striped mask. Scale bars: 200 μm.

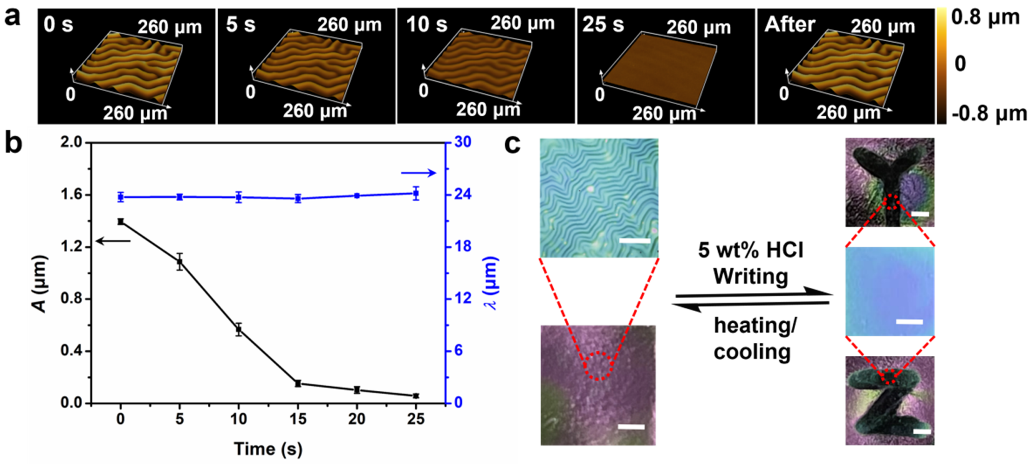

Supplementary Fig. 19. Evolution process of the pattern upon HCl vapor treatment. a LCSM images of wrinkles when the wrinkled samples were exposed to 56.4 ppm HCl vapor for 0, 5, 10, 15, and 25 s, and subsequently reheated to release the HCl. **b** The corresponding A (black line) and λ (blue line) of the wrinkles, error bars represent the standard deviations of three independent data. **c** Optical images (lower left, top right and lower right) of letters "Y" and "Z" on PDMS under natural light written by a writing brush with 5 wt.% HCl. Scale bars: 2 mm. The corresponding 2D LSCM images (top left and right middle) exhibiting characteristic wrinkled and flat surfaces. Scale bars: 100 μm. Source data are provided as a Source Data file.

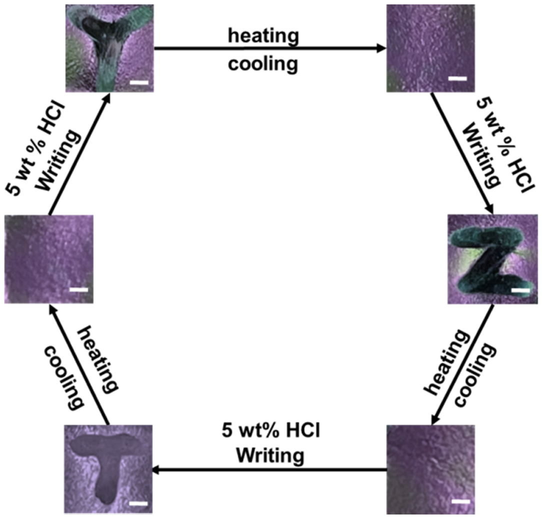

Supplementary Fig. 20. Optical images of letters on PDMS. The PDMS under natural light illustrating the application of the reversible pattern in response to acid for smart displays. Scale bars: 2 mm.

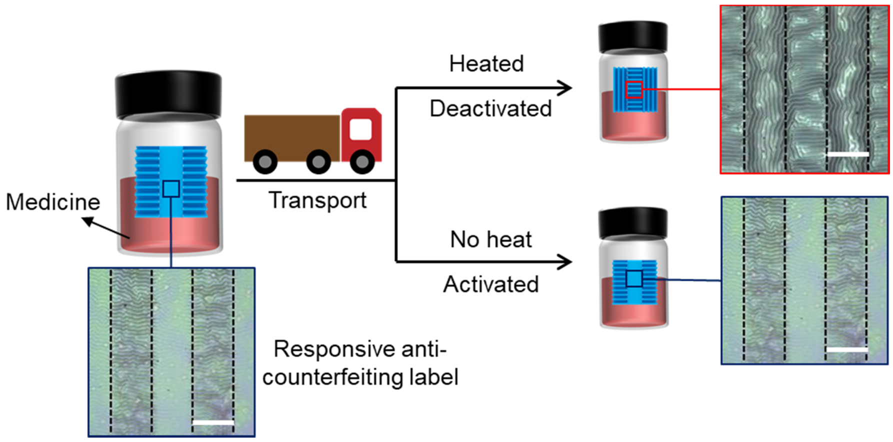

Supplementary Fig. 21. Application of transformable wrinkles. The wrinkle patterns serving as thermal-responsive anti- counterfeiting labels for medicine. Scale bars: 200 μm.

Supplementary Table

Supplementary Table 1. The control samples with different components of the top film and the detailed comparison. 1 PPy-Ba-St. **2** PPy-Ba-St@Phenyl disulfide. **3** PPy-Ba-St@H2MDB. **4** PPy-Ba-St@DTNB.

Number	Sample	Wrinkle	Erasure	Reverse
1	PPy-Ba-St	-	-	-
2	PPy-Ba-St@Phenyl disulfide	-	-	-
3	PPy-Ba-St@H2MDB	√	-	-
4	PPy-Ba-St@DTNB	√	√	√

Supplementary Discussion

To calculate the values of the stresses in the film-substrate systems, the following parameters were used: the Young's modulus of the substrate E_s=2 MPa, Poisson's ratio of the substrate υ_s=0.50 , Poisson's ratio of the film in the exposed and unexposed areas $\upsilon_{fe} = \upsilon_{fu}$=0.50, the thermal expansion coefficients of the substrate and film α_s-$\alpha_f \approx \alpha_f \approx 300\times10^{-6}$/°C, and the thicknesses of the substrate, the film in the exposed area, and the film in the unexposed area h_s=0.4 cm, h_f = 220 nm, respectively.

Supplementary Note 1. Stress analysis of the exposed area

Before exposed by the 365 UV light, the samples are heated and 2D disordered wrinkles are triggered (Fig. 3a). The initial thermal stresses induced by different thermal expansion coefficients between the surface thin film and PDMS substrates can be given by

$$\sigma_0 = \frac{E_{f1}\left(\alpha_s - \alpha_f\right)\Delta T}{\left(1-\upsilon_f\right)} \tag{S1}$$

where E_{fl} denotes the Young's modulus of the film before 365nm UV exposure, α_s and α_f are the thermal expansion coefficients of soft substrate and stiff thin film, respectively. ΔT denotes the temperature difference of the heat treatment. υ_f is the Poisson's ratio of surface film.

Before UV exposure

As shown in Fig. 3b, the polymer chains in networks before UV exposure are subjected to the isotropical thermal compressive stress σ_0, the average length of polymer chains along the 1st and 2ed directions in deformed random networks can be given by

$$r_1 = \theta_1{}^{(1)} r_0 \tag{S2a}$$

$$r_2 = \theta_2{}^{(1)} r_0 \tag{S2b}$$

where the average magnitude of end to end vectors in the un-deformed state r_0, $\theta_1{}^{(1)}$ and $\theta_2{}^{(1)}$ are the principal compression ratio along the 1st and 2ed directions.

Within a small deformation range of thermal strain, the stress-stretch relations can be give by [1]

$$\Delta\sigma(t) = kT_a N\nu(\frac{r_0}{Nb})^2[\theta^2 - \frac{1}{\theta^2}] \tag{S3}$$

where k the Boltzmann constant and T_a the absolute temperature.

The elastic modulus of the polymer network can be obtained by the initial slope of the macroscopic stress-stretch relation as follows

$$E = \lim_{\theta \to \theta_1} \frac{d(\Delta\sigma)}{d\theta} \quad \text{(S4)}$$

Substituting Supplementary Eq. (3) into Supplementary Eq. (4), yeilds

$$E_1 = 2(kT_a N\nu)\left(\frac{r_0}{Nb}\right)^2\left[\theta_1 + \frac{1}{{\theta_1}^3}\right] \quad \text{(S5)}$$

After UV exposure

After UV exposure, the photoinduced disulfide bond exchange can make deformed polymer chains return to the initial equilibrium position, which is similar to the process of restoring the compressed spring to its equilibrium point (Fig. 3b). The stress relaxation process can be depicted through two-step modeling including free state without boundary constraints (Fig. 3b ii) and constraint state by boundary stresses (Fig. 3b iii).

(1) Free state without boundary constraints

When the photosensitive surface film is under free state without boundary constraints, the deformed polymer chains freely restore to the initial equilibrium position at constant disulfide bond exchange rate, the disulfide bond ratio without exchange satisfies the following relationship as

$$\frac{d\eta}{dt} = -r_n\eta \quad \text{(S6)}$$

where η is the disulfide bond ratio without exchange, r_n is the photoinduced S-S bond exchange rate.

Solving Supplementary Eq. (6), yeilds

$$\eta = Ce^{-r_n t} \quad \text{(S7)}$$

The thermal stress will dynamically relax with the disulfide bond exchange, therefore, the compression ratio also changes with exposure time, which can be given by

$$\theta(\mathrm{t}) = \theta_1 + (1-\theta_1)(1-\eta) \quad \text{(S8)}$$

Substituting Supplementary Eq. (7) into Supplementary Eq. (8), yeilds

$$\theta(\mathrm{t}) = \theta_1 + (1-\theta_1)(1-Ce^{-r_n t}) \quad \text{(S9)}$$

By combining Supplementary Eqs. (9) and (3), the time-varying residual thermal stress can be expressed as

$$\Delta\sigma(t) = kT_a N\nu(\frac{r_0}{Nb})^2[\theta^2(t) - 1/\theta^2(t)] \quad \text{(S10)}$$

After full UV exposure, the deformed polymer chains restore to the initial equilibrium position and the the average length of polymer chains along the 1st and 2ed directions in deformed random networks (Fig. 3bii) can be given by

$$r_1 = r_2 = r_0 \quad \text{(S11)}$$

where $\theta_1^{(1)} = \theta_2^{(1)} = 1$.

As inferred from Supplementary Eq. (11), the stress in the polymer chains $\Delta\sigma = 0$, which indicates that the stress in surface film in free state can be eliminated after full UV exposure. The elastic modulus of the polymer network at $\theta_1^{(1)} = \theta_2^{(1)} = 1$ can be defined as E_0.

(2) Constraint state by boundary stresses

As shown in Fig. 3d and Fig. 3f, $\sigma_x = 14.17$ MPa, $\sigma_y = 78.33$ MPa, then $\theta_1^{(2)} = 0.9945$, $\theta_2^{(2)} = 0.9697$, which indicates that the polymer network under the constraint of boundary stress can be slight compressed after photoinduced S-S bond exchange. Moreover, as shown in Supplementary Fig. 16, the normalized elastic moduli of the polymer network along x and y directions can be calculated as $E_x/E_0 = 1.0249$, $E_y/E_0 = 1.0529$, where E_0 is the initial elastic modulus of the undeformed polymer network. It is indicated that the photoinduced disulfide bond exchange under the constraint of boundary stress can result in lead to a very small increase in elastic modulus of surface film. The increment of elastic modulus is too small to be characterized (Supplementary Fig. 13).

Supplementary Note 2. Stress analysis of the unexposed area

For the unexposed area, surface film remains homogeneous due to no disulfide bond exchange. As shown in Fig. 3e, the boundaries of the unexposed area are the exposed area. 365nm UV exposure can cause disulfide bond exchange and stress relaxation of the exposed area, which results in stress reduction at the boundary ($x=\pm W/2$) and further triggers 1D stress relaxation of the unexposed area. In light of the experimental configurations, the soft boundary conditions of the unexposed area can be given by

$$\bar{E}_{f1}\frac{du_x}{dx}+\sigma_0=\sigma_1 \quad \left(x=\pm\frac{W}{2}\right) \tag{S12}$$

A finite width soft-boundary thin film is bonded to a compliant PDMS substrate with thickness h_s, where the length, width and thickness of thin film are L, W and h_f, respectively. The strains of the surface film can be expressed as

$$\varepsilon_x=\frac{du_x}{dx},\ \ \varepsilon_y=0,\ \ \varepsilon_{xy}=0 \tag{S13}$$

where ε_x, ε_y, ε_{xy} are the strain components in the *x, y* and *xy* directions, respectively.

According to the constitutive relation of the top film, the stresses in the thin film can be given by

$$\sigma_x=\sigma_0+\bar{E}_{f1}\frac{du_x}{dx} \tag{S14a}$$

$$\sigma_y=\sigma_0+\upsilon_{f1}\bar{E}_{f1}\frac{du_x}{dx} \tag{S14b}$$

Due to soft boundaries $(x=\pm W/2)$, thermal-induced residual stress σ_0 can relax in the *x* direction, which can cause a non-uniform stress distribution along with the width of the film. For 1D relaxation, the stress distribution in the *x* direction can be expressed as

$$\frac{d\sigma_x}{dx}=\frac{T_x}{h_f} \tag{S15}$$

where the shear traction at the interface between the film and the substrate

$T_x = \mu_R u_x / h_s$, μ_R denotes the elastic shear modulus of substrate at the rubbery limit.

Substituting Supplementary Eq. (14a) into Supplementary Eq. (15) yields

$$\frac{d^2 u_x}{dx^2} = \frac{\mu_R u_x}{\bar{E}_{f1} h_s h_f} \quad \text{(S16)}$$

Supplementary Eq. (16) is solved with the boundary conditions Supplementary Eq. (12), and the stress distributions can be obtained as

$$\sigma_x = \sigma_0 \left[1 - \left(1 - \frac{\sigma_1}{\sigma_0} \right) \frac{\cosh(x/\lambda_l)}{\cosh(W/2\lambda_l)} \right] \quad \text{(S17a)}$$

$$\sigma_y = \sigma_0 \left[1 - \upsilon_{f1} \left(1 - \frac{\sigma_1}{\sigma_0} \right) \frac{\cosh(x/\lambda_l)}{\cosh(W/2\lambda_l)} \right] \quad \text{(S17b)}$$

where the term of '$(1-\sigma_1/\sigma_0)$' is induced by soft boundary conditions, and the shear-lag length $\lambda_l = \sqrt{\bar{E}_{f2} h_s h_f / \mu_R}$.

As indicated in Supplementary Eq. (17), the 1D relaxation along x direction can cause non-uniform stress distribution. As shown in Fig. 3f, when $x < -64.3$ μm or $x > 64.3$ μm , $\sigma_y > \sigma_c > \sigma_x$, which indicates that the 1D ordered wrinkles along y direction will be triggered before the wrinkles along x direction. Therefore, the theoretical results can perfectly explain the experimental results (Fig. 3a).

Supplementary Note 3. Stress analysis of surface film/substrate after heating/cooling treatment

When the samples are heated, the the average length of polymer chains along the 1st and 2^{ed} directions in deformed random networks are stretched, which can be given by

$$r_1 = \theta_1^{(3)} r_0 \quad \text{(S18a)}$$

$$r_2 = \theta_2^{(3)} r_0 \quad \text{(S18b)}$$

When the samples are cooled, the the average length of polymer chains along the 1st and 2^{ed} directions in deformed random networks are compressed, which can be

given by

$$r_1 = {\theta_1}^{(4)} r_0 \tag{S19a}$$

$$r_2 = {\theta_2}^{(4)} r_0 \tag{S19b}$$

Because of the anisotropic thermal expansion in PDMS and the heterogeneous boundary along *x*/*y* direction in the exposed area, we can obtain that ${\theta_1}^{(4)} = {\theta_1}^{(2)} > {\theta_2}^{(4)} = {\theta_2}^{(2)}$.

Stress analysis of the exposed area

For heterogeneous surface film in the exposed area, the elastic moduli of the polymer network satisfy the following relationship

$$E_0 < E_x < E_y \tag{S20}$$

According to $\sigma^c = \bar{E}_f \left(3\bar{E}_s / \bar{E}_f\right)^{2/3} / 4$, the critical stress in *y* direction is larger than the critical stress in *x* direction, which indicates that the wrinkles along *x* direction can be triggered before the wrinkles along *y* direction in the exposed area (Fig. 3a).

$$\sigma_y^c > \sigma > \sigma_x^c \tag{S21}$$

Stress analysis of the unexposed area

For the unexposed area, the elastic modulus of surface film is smaller than the boundary of exposed surface film

$$E_{ue} = E_0 < E_e = E_x \tag{S22}$$

As indicated by Supplementary Eq. (22), the residual thermal stress is difficult to release at the boundary (*x*=±*W/2*). Therefore, the stresses in the unexposed surface film satisfy the following relationship

$$\sigma_x > \sigma_c > \sigma_y \tag{S23}$$

According to Supplementary Eq. (23), the wrinkles parallel to the boundary (*x*=±*W/2*) can be triggered first in the unexposed area (Fig. 3a, e, f).

Supplementary references

1 Alamé, G. & Brassart, L. Relative contributions of chain density and topology to the elasticity of two-dimensional polymer networks. *Soft matter* **15**, 5703-5713 (2019).
